# Trends in Weight Gain Among Breastfed Infants Versus Bottle-Fed Infants at a Tertiary Care Hospital in Karachi, Pakistan

**DOI:** 10.7759/cureus.23459

**Published:** 2022-03-24

**Authors:** Geeta Bai, Arit Parkash, Vikash Kumar, Meena Kumari, Satvantee Kumari, Kirpal Das

**Affiliations:** 1 Department of Pediatrics, National Institute of Child Health, Karachi, PAK; 2 Department of Pediatric Gastroenterology and Hepatology, National Institute of Child Health, Karachi, PAK; 3 Department of Internal Medicine, The Brooklyn Hospital Center, New York, USA; 4 Department of Pediatircs, Jinnah Postgraduate Medical Centre, Karachi, PAK; 5 Department of Obstetrics and Gynecology, Jinnah Postgraduate Medical Centre, Karachi, PAK; 6 Department of Internal Medicine, Khairpur Medical College, People’s University of Health Sciences for Women, Khairpur, PAK

**Keywords:** exclusive breastfeeding, pakistan, weight gain, monthly income, bottle feeding

## Abstract

Background

Infants need to be exclusively breastfed up to six months of age, and breastfeeding should be continued up to two years of age along with complementary food. In Pakistan, the majority of newborns are not exclusively breastfed. This study was done to compare weight gain between breastfed infants and non-breastfed infants at a tertiary care hospital in Karachi, Pakistan.

Methodology

This observational cohort study was conducted at the well-baby clinic and vaccination center of the National Institute of Child Health, Karachi, Pakistan, from January 2021 to December 2021. A total of 360 normal term babies (180 in each group) with age below 11 months on either exclusively breastfeeding or other milk feed were included. Data were collected by the duty senior staff nurse of the well-baby clinic and monitored on daily basis by the researchers. The sociodemographic characteristics of mothers of breastfed and non-breastfed babies and birth weight, length, and BMI Z scores of babies in both groups were compared.

Results

In a total of 360 babies, there were 192 (53.3%) boys and 168 (46.7%) girls. Overall, the mean maternal age was calculated to be 28.1±6.2 years, ranging between 18 and 37 years. The employment status of mothers (p=0.0117) and monthly income of parents (p=0.0388) were significantly different between groups. The mean weight gain in the exclusively breastfeeding group was 4.0±0.5 kg between the first and fifth visit (final visit) in comparison with 4.5±0.5 kg in the non-breastfeeding group (p<0.0001).

Conclusion

Non-breastfed babies gained significantly more weight in comparison with exclusively breastfed babies. More multicenter trials involving a large proportion of populations are needed to further verify the findings of the present study.

## Introduction

Infants should be exclusively breastfed until six months of age, and breastfeeding should be continued until two years of age along with complementary food. Unfortunately, in Pakistan, exclusive breastfeeding rates, until six months of age, are calculated to be around 38%, which means that more than half of newborns are not exclusively breastfed [1]. On the other hand, non-breastfed infants have been observed to have increased weight gain and higher BMI values, which may lead to obesity later in life [2]. Moreover, an increased number of adipocytes in infancy predispose to obesity in childhood and in adults [3,4]. Birth weight and growth rate during infancy are important factors determining the predisposition of the development of obesity [5-7].

Ebina and Kashiwakura focused on weight gain in the first month of life in their study and found that there was no difference in exclusively breastfed or formula-fed infants [8]. Azad et al. in a recent study described the risk of being overweight to be threefold higher in non-breastfed as compared with exclusively breastfed infants (8.3% versus 2.4%) [9]. In a recent cross-sectional survey from Pakistan, no significant difference was found in the weights of breastfed and non-breastfed infants [10].

In a developing country like Pakistan, there is a significant burden of not only undernourished children but also obesity, which is also on the rise, making both types of malnutrition prevalent [11]. We have lower rates of exclusive breastfeeding [1]. The first six months of life is a period where, in most infants, only milk is recommended. There are some inconsistencies and conflicts in exposure (non-breastfed) and outcome (excessive weight gain) previously, and no prospective study is reported from Pakistan. There is a need to assess the growth rates of breastfed and non-breastfed babies and to compare with normal growth rates on WHO centile charts (gender-specific) to know the trends of weight gain. This study was done to compare the weight gain between breastfed infants and non-breastfed infants at a tertiary care hospital in Karachi. We hypothesized that non-breastfed infants gain excessive weight as compared with exclusively breastfed infants.

## Materials and methods

Study design

The present study is an observational cohort study.

Place and duration of the study

This study was conducted at the well-baby clinic and vaccination center of the National Institute of Child Health, Karachi, Pakistan, from January 2021 to December 2021.

Inclusion criteria

We included babies aged less than one month on either exclusive breastfeeding or any other milk feed. We considered normal infants, born on term gestational age (>37 weeks).

Exclusion criteria

Infants developing diseases during the study period (such as neonatal hepatitis, neonatal sepsis, hypothyroidism, and hemolytic anemia), which affect weight gain, were excluded. Babies with congenital heart disease, congenital gastrointestinal obstructions, stenosis, or malformations and syndromic babies were also not included.

Sample size

The sample size was calculated at a confidence level of 90%, power of 80%, and ratio in non-breastfed as 8.3% and breastfed as 2.4%, and it turned out to be 360 (180 in each group).

Data collection

Approval from the Institutional Ethics Review Board (IERB-09/2021) of the National Institute of Child Health, Karachi, Pakistan, was acquired. Informed written consents were taken from the parents/guardians of all study participants. Non-probability consecutive sampling technique was adopted. Data were collected by the duty senior staff nurse of the well-baby clinic and monitored on daily basis by the researchers. The senior staff nurse was well trained in taking anthropometric measurements, and routine measurements were taken when babies came for vaccination at each visit. Data were collected from the well-baby clinic and vaccination center in the medical outpatient department of the National Institute of Child Health, Karachi, Pakistan. Mothers were asked about their feeding practice, either breastfeeding or bottle-feeding. Anthropometry (including weight in kg, length in cm (converted to meters), and BMI (kg/m^2^)) of babies at each visit were recorded at birth to the first week of age (when they came for the first dose of vaccine), second visit at six weeks to eight weeks of age (for the second dose of vaccine), third visit at 10 weeks to 12 weeks of age (for the third dose of vaccine), fourth visit at 14 weeks to 16 weeks of age (for the fourth dose of vaccine), and final visit of the baby at 23 weeks to 25 weeks of age (they were specially asked for that last visit). The contact numbers of mothers were taken by the nurse to remind them of the next due visit for their baby to ensure compliance in follow-up. Complete assurance of secrecy of study data and the personal information of the mother were guaranteed. Weight was measured using an infant weight machine, length was be measured using an infantometer, and head circumference was measured using a measuring tape. The same machines were used for follow-up measurements. All study data were collected on customized proforma.

Exclusively breastfed infants are those infants who were on exclusive breastfeeding for the initial six months of life or infants who were delivered by cesarean section and took formula feed for the initial one week, followed by exclusive breastfeeding for the rest of the six months. Bottle-fed (non-breastfed) infants are those children who had any food or liquid including nonhuman milk and formula [15]. Weight gain was described as BMI for infants according to the WHO gender-specific charts [16-18]: wasting, <3rd; normal, ≥3rd and ≤85th; at risk of overweight, >85th and ≤97th; and overweight, >97th. Z score was calculated for weight-for-length (WLZ) [11,12].

Both exposed (bottle-fed) and non-exposed (breastfed) groups were followed for outcome (excessive weight gain plotted on the WHO gender-specific growth charts for BMI of infants). Both groups were recruited at the first vaccination dose (first week of life) and followed for six months of age. Measurement and observer bias were controlled by proper training and monitoring of the staff nurse (data collector). Strict following of standard operational definitions was done, and researchers made visits to see at least 10% of cases for quality check. Data were kept confidential, and only the nurse and researchers had access to the data.

Statistical analysis

Data were entered and analyzed in Statistical Package for the Social Sciences (SPSS) version 26.0 (IBM Corporation, Armonk, NY, USA). Qualitative variables were summarized as percentages and quantitative variables as mean and standard deviations (SDs). The sociodemographic characteristics of mothers of breastfed and non-breastfed babies and birth weight, length, and BMI Z scores of babies in both groups were compared using the chi-square test for categorical variables and t-test for quantitative variables. The mean weights of both groups were calculated using paired sample t-test to see the difference, considering a p-value of <0.05 as significant.

## Results

In a total of 360 babies, there were 192 (53.3%) boys and 168 (46.7%) girls. Overall, the mean maternal age was calculated to be 28.1±6.2 years, ranging between 18 and 37 years. There were 277 (76.9%) mothers who had ≤2 children. The educational status of 122 (33.9%) mothers was illiterate. There were 47 (13.1%) mothers who were employed. The monthly parents’ income of 158 (43.9%) babies was low. Table 1 shows the comparison of the characteristics of mother-infant pairs. It was observed that the employment status of mothers (p=0.0117) and monthly income of parents (p=0.0388) were significantly different in both study groups.

**Table 1:** Characteristics of Mother-Infant Pairs * A Chi-square test was used. ** An independent sample t-test was used.

Characteristics		Exclusively Breastfeeding (n=180)	Non-breastfeeding (n=180)	P-value
Baby’s Gender	Male	98 (54.4%)	94	0.6726*
	Female	82 (45.6%)	86	
Maternal Age in Years (Mean±SD)		28.4±6.1	27.8±6.5	0.3671**
Number of Children	≤2	116 (64.4%)	111	0.5851*
	>2	64 (35.6%)	69	
Educational Status of Mothers	Illiterate	68 (37.8%)	54	0.1190*
	Literate	112 (62.2%)	126	
Employment Status of Mothers	Employed	18 (10%)	29 (16.1%)	0.0117*
	Housewife	33 (90%)	127 (83.9%)	
Monthly Income of Parents	Low	80 (44.4%)	78 (43.3%)	0.0388*
	Middle	79 (43.9%)	64 (35.6%)	
	High	21 (11.7%)	38 (21.1%)	

Table 2 shows the comparison of weight, length, and BMI among babies of both study groups during the course of the study. It was noted that the mean net weight gain in the exclusively breastfeeding group was 4±0.5 kg between the first and the fifth visit (final visit) in comparison with 4.5±0.5 in the non-breastfeeding group, and the difference was noted to be statistically significant (p<0.0001). The mean body length was calculated to be 65.8±1.5 cm in the exclusively breastfeeding group in comparison with 64.5±1.6 in the non-breastfeeding group; the difference was noted to be statistically significant (p<0.0001). A statistically significant difference also existed in the BMI Z scores of exclusively breastfed and non-breastfed babies, and it was found to be -0.8+0.6 versus 0.3+0.5 (p<0.0001).

**Table 2:** Comparison of Weight, Length, and BMI Among Babies of Both Study Groups During the Course of the Study A paired sample t-test was used for the comparison of data.

Outcomes	Groups	Visits				
		First (Mean+SD (n))	Second (Mean+SD (n))	Third (Mean+SD (n))	Fourth (Mean+SD (n))	Fifth (Mean+SD (n))
	Exclusively Breastfeeding	2.9±0.5 (180)	3.8±0.6 (171)	4.8±0.7 (154)	6.0±1.1 (141)	6.9±1.0 (126)
	Non-breastfeeding	2.8±0.4 (180)	4.0±0.7 (166)	5.3±0.8 (152)	6.4±0.9 (138)	7.3±0.9 (117)
	P-value	0.4025	0.0051	<0.0001	0.0010	0.0012
Length (cm)	Exclusively Breastfeeding	49.1±0.7 (180)	52.7±1.0 (171)	56.0±1.1 (154)	61.2±1.3 (141)	65.8±1.5 (126)
	Non-breastfeeding	49.2±0.6 (180)	52.6±0.9 (166)	55.8+1.2 (152)	60.8±1.1 (138)	64.5±1.6 (117)
	P-value	0.1465	0.3357	0.1295	0.0060	<0.0001
BMI Z Score	Exclusively Breastfeeding	-0.9±0.2 (180)	-0.2±0.4 (171)	-0.1±0.2 (154)	-0.1±0.4 (141)	-0.8+0.6 (126)
	Non-breastfeeding	-1.1+0.3 (180)	0.3±0.4 (166)	1.4±0.5 (152)	0.5±0.4 (138)	0.3+0.5 (117)
	P-value	<0.0001	0.0224	<0.0001	<0.0001	<0.0001

## Discussion

In this study, we aimed to determine weight gain patterns from 0 to 6 months of life among children who were exclusively breastfed and non-breastfed. One of the major strengths of this study is that we not only opted for weight and length comparisons of the babies during the course of this study but also calculated and compared BMI Z scores. Although the BMI Z scores in both study groups at the end of the study were found to be in the healthy range, it was observed that non-breastfeeding was associated with significantly more weight gain in comparison with exclusive breastfeeding. According to a meta-analysis, there was a significant association between non-breastfeeding and rapid weight gain during infancy [13]. Researchers have also suggested that non-breastfeeding might not be directly linked with rapid weight gain during infancy, but rapid weight gain could be a result of the variation of protein in different formula feeds and due to physiological and behavioral aspects related to the babies and their caregivers [14,15]. A study from Italy correlates well with the findings of the present study, where Agostoni et al. revealed that babies fed with formula during the first 12 months of life gained significantly more weight in comparison with those who were exclusively breastfed [16]. A study by Huang et al. noted that early feeding of higher volumes of formula was linked with higher body weight/obesity in the later infancy period [17]. The authors found that non-breastfed infants had 1.60 times more chance of higher body weight (1 SD<WLZ≤2 SD) in comparison with exclusively breastfed infants by the end of six months of age. The same study also reported that non-breastfed infants had 2.13 times higher chances of being overweight (WLZ>2 SD) when compared with exclusively breastfed infants by the age of 12 months. A systemic review and meta-analysis performed by Gale et al. shared that in comparison to breastfeeding, formula feeding was linked with altered body composition in the infancy period [18].

The possible mechanism behind more weight gain in non-breastfed babies is still not fully understood. Biological mechanisms regarding the unique attributes of breastmilk such as leptin and adiponectin might be helpful in regulating energy intake [19,20]. Babies might be spending more time latching and suckling while breastfeeding. It is also possible that mothers feeding their babies with exclusive breastfeeding might have learned feeding quantity and style. Researchers have reported that the duration and amount of bottle-feeding are usually dependent on the caregiver’s decision, such as what amount of milk is remaining in the bottle, or they often encourage the baby to finish the bottle [21,22]. Variations in the taste and the nutrients during breastfeeding might also be playing some role in providing physiological signals to the infant for stopping suckling. So, babies feeding with bottles might be gradually losing their abilities to self-regulate and might end up gaining more weight rapidly in comparison with the ones having exclusive breastfeeding.

Limitations of the study

Our study had some limitations as well. As this was a single-center study conducted in a specific region of Pakistan, our findings cannot be generalized for the overall population. We could not determine the possible association of dietary patterns, sedentary behaviors, or sleep duration. We also could not keep track of the number of feedings per day during the study period, which could have been important in relating to weight alterations during the first six months of life. Maternal nutritional status was not accounted for. As this was not a randomized study, there could have been a selection bias during enrollment for this study.

## Conclusions

Non-breastfed babies gained significantly more weight in comparison with exclusively breastfed babies. More multicenter trials involving a large proportion of populations are needed to further verify the findings of the present study.

## References

[1] (2022). World Health Organization: Breastfeeding gives babies the best possible start in life and breastmilk works like a baby’s first vaccine. http://www.emro.who.int/pak/pakistan-news/breastfeeding-gives-babies-the-best-possible-start-in-life-and-breastmilk-works-like-a-babys-first-vaccine.html.

[2] Bell KA, Wagner CL, Feldman HA, Shypailo RJ, Belfort MB (2017). Associations of infant feeding with trajectories of body composition and growth. Am J Clin Nutr.

[3] Pirilä S, Saarinen-Pihkala UM, Viljakainen H, Turanlahti M, Kajosaari M, Mäkitie O, Taskinen M (2012). Breastfeeding and determinants of adult body composition: a prospective study from birth to young adulthood. Horm Res Paediatr.

[4] Koontz MB, Gunzler DD, Presley L, Catalano PM (2014). Longitudinal changes in infant body composition: association with childhood obesity. Pediatr Obes.

[5] Demerath EW, Choh AC, Czerwinski SA (2007). Genetic and environmental influences on infant weight and weight change: the Fels Longitudinal Study. Am J Hum Biol.

[6] (2010). World Health Organization: Noncommunicable diseases: Childhood overweight and obesity. http://www.who.int/dietphysicalactivity/childhood/en.

[7] Mansoori N, Nisar N, Shahid N, Mubeen SM, Ahsan S (2018). Prevalence of obesity and its risk factors among school children in Karachi, Pakistan. Trop Doct.

[8] Ebina S, Kashiwakura I (2013). Relationship between feeding modes and infant weight gain in the first month of life. Exp Ther Med.

[9] Azad MB, Vehling L, Chan D (2018). Infant feeding and weight gain: separating breast milk from breastfeeding and formula from food. Pediatrics.

[10] Hussain Z, Khan N (2017). Assessment of the nutritional status of bottle-fed infants and the prevalence of infections, allergy and diarrhea among bottle fed infants and its comparison with exclusively breast fed infants aged 0-6 months. J Pediatr Neonatal Care.

[11] Furlong KR, Anderson LN, Kang H (2016). BMI-for-age and weight-for-length in children 0 to 2 years. Pediatrics.

[12] (2022). https://www.who.int/childgrowth/standards/chts_bfa_boys_z/en/.

[13] Appleton J, Russell CG, Laws R, Fowler C, Campbell K, Denney-Wilson E (2018). Infant formula feeding practices associated with rapid weight gain: a systematic review. Matern Child Nutr.

[14] Li R, Magadia J, Fein SB, Grummer-Strawn LM (2012). Risk of bottle-feeding for rapid weight gain during the first year of life. Arch Pediatr Adolesc Med.

[15] Mihrshahi S, Battistutta D, Magarey A, Daniels LA (2011). Determinants of rapid weight gain during infancy: baseline results from the NOURISH randomised controlled trial. BMC Pediatr.

[16] Agostoni C, Grandi F, Giannì ML, Silano M, Torcoletti M, Giovannini M, Riva E (1999). Growth patterns of breast fed and formula fed infants in the first 12 months of life: an Italian study. Arch Dis Child.

[17] Huang J, Zhang Z, Wu Y (2018). Early feeding of larger volumes of formula milk is associated with greater body weight or overweight in later infancy. Nutr J.

[18] Gale C, Logan KM, Santhakumaran S, Parkinson JR, Hyde MJ, Modi N (2012). Effect of breastfeeding compared with formula feeding on infant body composition: a systematic review and meta-analysis. Am J Clin Nutr.

[19] Houseknecht KL, McGuire MK, Portocarrero CP, McGuire MA, Beerman K (1997). Leptin is present in human milk and is related to maternal plasma leptin concentration and adiposity. Biochem Biophys Res Commun.

[20] Singhal A, Farooqi IS, O'Rahilly S, Cole TJ, Fewtrell M, Lucas A (2002). Early nutrition and leptin concentrations in later life. Am J Clin Nutr.

[21] Nelson AM (2006). A metasynthesis of qualitative breastfeeding studies. J Midwifery Womens Health.

[22] Arnold L (1999). Recommendations for collection, storage and handling of a mother’s milk for her own infant in the hospital setting. HMBANA.

